# Abnormal phase separation of biomacromolecules in human diseases

**DOI:** 10.3724/abbs.2023139

**Published:** 2023-07-21

**Authors:** Songhao Zhang, Gaofeng Pei, Boya Li, Pilong Li, Yi Lin

**Affiliations:** 1 State Key Laboratory of Membrane Biology Tsinghua University-Peking University Joint Centre for Life Sciences School of Life Sciences Tsinghua University Beijing 100084 China; 2 IDG/McGovern Institute for Brain Research at Tsinghua University Beijing100084 China; 3Beijing Frontier Research Center for Biological Structure Tsinghua University Beijing 100084 China

**Keywords:** membrane-less organelles, developmental disorder, tandem repeat disorder, cancer, infectious diseases, abnormal phase separation

## Abstract

Membrane-less organelles (MLOs) formed through liquid-liquid phase separation (LLPS) are associated with numerous important biological functions, but the abnormal phase separation will also dysregulate the physiological processes. Emerging evidence points to the importance of LLPS in human health and diseases. Nevertheless, despite recent advancements, our knowledge of the molecular relationship between LLPS and diseases is frequently incomplete. In this review, we outline our current understanding about how aberrant LLPS affects developmental disorders, tandem repeat disorders, cancers and viral infection. We also examine disease mechanisms driven by aberrant condensates, and highlight potential treatment approaches. This study seeks to expand our understanding of LLPS by providing a valuable new paradigm for understanding phase separation and human disorders, as well as to further translate our current knowledge regarding LLPS into therapeutic discoveries.

## Introduction

Membrane-less organelles (MLOs) formed through liquid-liquid phase separation (LLPS), also known as biomolecular condensates, participate in various biological processes and are crucial for human health [ 1, 2] . These MLOs play vital roles in fundamental processes including heterochromatin formation [3], nucleocytoplasmic transport [ 4, 5] , nucleolus formation [6], transcription hub formation [ 7, 8] , innate immunity [9] and resistance to stresses [ 10‒ 12] . Nevertheless, the condensate may be disturbed or inclined to slowly transition into solid-like states due to various factors, including genetic mutations, diminished protein quality control, and impaired cellular transportation mechanisms. The aberrant transition of condensates is pathological and causatively associated with a variety of human diseases [ 13, 14] . This article mainly investigates a few different categories of diseases. In developmental disorders, the three major categories of LLPS-associated pathogenic processes are loss of function, gain of function, and gain of toxicity. Tandem repeat disorders (TRDs) can be classified based on the location of the repeating sequence and the production of aberrant RNA or proteins. In cancer, the aberrant phase separation can affect transcriptional regulation, signal transduction, or protein degradation. Furthermore, virus and host cells have a special connection in that cells use phase separation to resist viral infection, while viruses harness phase separation to complete infection. We will also explore the primary and possible LLPS mechanisms of pathogenesis under different conditions. This study aims to offer a thorough comprehension of the role of LLPS in various human diseases and to discuss potential points for therapeutic intervention.

## Developmental Disorders with Aberrant Phase Separation

Developmental disorders are diseases that entail deviations from normal development and often manifest during early childhood, of which, intellectual disability (ID) and autism are two examples [15]. Recent research has shown that liquid-liquid phase separation (LLPS) is implicated in a wide range of developmental and differentiation processes [ 16, 17] . For instance, the protein FXR1 engages in LLPS to retain non-translating mRNA and recruits translational machinery to activate target translation, which is vital in the process of spermiogenesis [18]. Similarly, the phase separation of both transcription factor SOX9 and chromatin modulator CBX2 is essential for testicular development [ 19, 20] . In this part, we will review recent studies on human developmental diseases that are associated with LLPS. These LLPS-associated pathogenic mechanisms can generally be categorized into three main categories: loss of function, gain of function, and gain of toxicity ( Table 1).

**Table:** **Table 1** The pathological mutations of phase separated-protein on developmental disorders

Disease	Associated protein	Mutation	Pathological type	Mechanism	Reference
Congenital dilated cardiomyopathy	RBM20	Arg636Ser	Gain of function	The mutation promotes RBM20 to transfer from the nucleus to cytoplasm and form liquid-like granules, which dock at myofibril Z-discs to disrupt the actin cytoskeleton of cardiomyocytes, further inducing congenital dilated cardiomyopathy.	[21]
Developmental delay/intellectual disability	DDX3X	Leu556Ser	Gain of toxicity	The mutation results in DDX3X misfolding and self-aggregation, transferring LLPS to solid-like condensates, which sequestrates the healthy DDX3X and impairs cell viability.	[22]
Rett syndrome	MeCP2	Arg168Ter, Arg255Ter, Arg270Ter, Arg294Ter, Pro389Ter; Arg133Cys, Thr158Met, Pro225Arg, Arg306Cys, Pro322Leu	Loss of function	The mutations interfere with its capacity to form LLPS, which decreases MeCP2 mutant and its cofactor partitioning into heterochromatin condensates, causing altered chromatin architecture and other cellular abnormalities linked to Rett syndrome.	[23]
Noonan syndrome	SHP2	Asp61Gly, Glu76Ala, Glu76Lys	Gain of function	The SHP2 mutations trigger the closed to open conformation transition, which results in an electrostatic contact and promotes the formation of condensates, further recruiting WT SHP2 to encourage MAPK activation.	[24]
Leopard syndrome	SHP2	Tyr279Cys, Gly464Ala, Thr468Met, Arg498Leu, Gln506Pro	Gain of function	The SHP2 mutations trigger the closed to open conformation transition, which results in an electrostatic contact and promotes the formation of condensates, further recruiting WT SHP2 to encourage MAPK activation.	[24]
Kabuki Syndrome	MLL4	Gln4092Ter	Loss of function	The mutation impairs MLL4’s capacity for LLPS and reduces transcriptional condensate formation, which alters the balance between transcriptional and PcG condensates, further changing the nuclear architecture.	[25]
Autism spectrum disorders	CTTNBP2	Met120Ile, Arg533Ter	Loss of function	The CTTNBP2 mutant forms smaller and fewer condensates in dendritic spines than the WT, which impairs social interactions in mutant mice.	[ 26‒ 28]
Ulnar-mammary syndrome	TBX3	Leu143Pro, Tyr149Ser, Ser190Arg, Gln475Ter	Loss of function	The mutation affects TBX3’s LLPS capacity to drive appropriate transcriptional regulation of important neuropeptides (TAC3 and KISS1) in KNDy neurons, which further impairs the identity of KNDy neurons and delays the beginning of puberty.	[29]

### Loss of function

**Figure 1:**
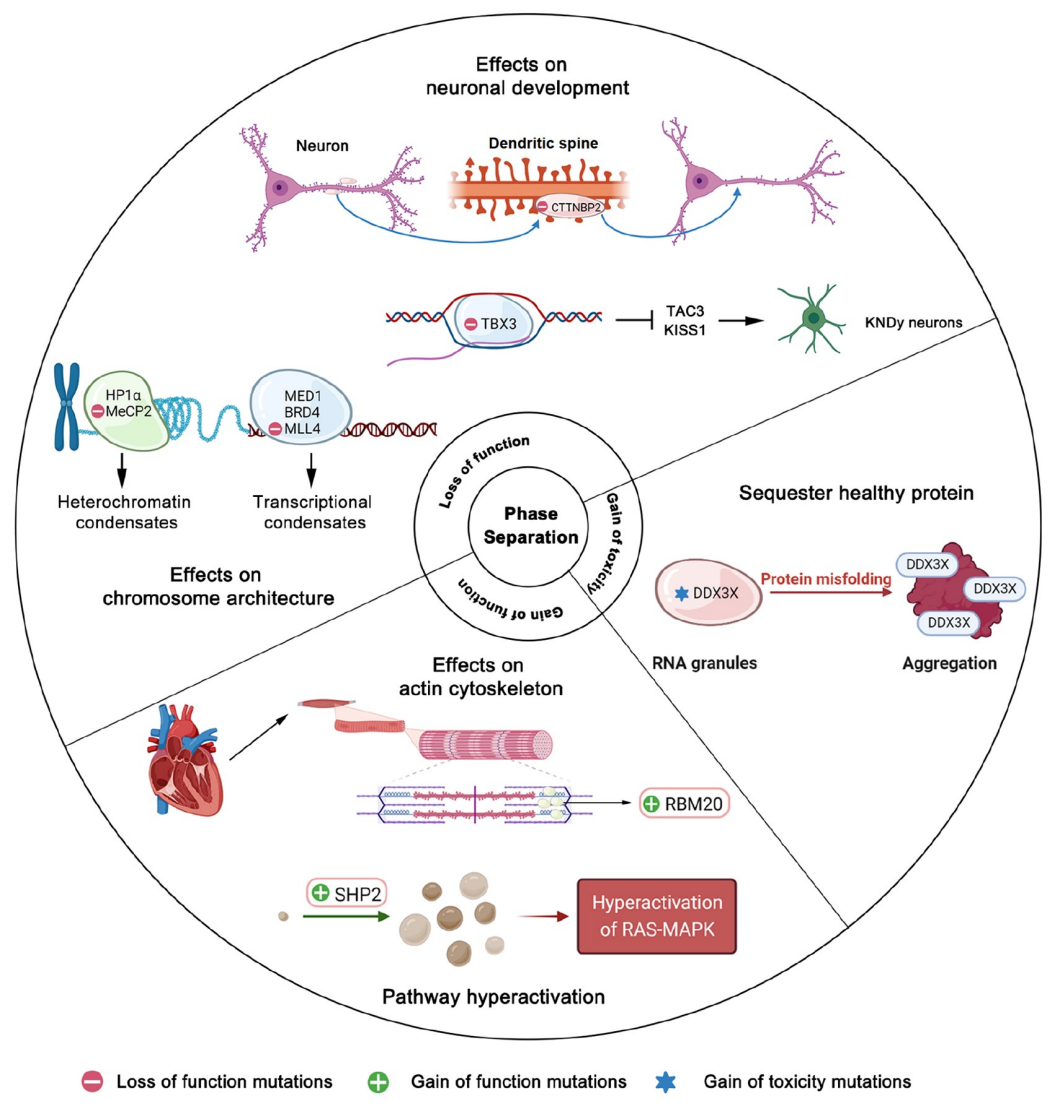
Figure 1 Dysregulated phase separation by pathological mutations in developmental disorders Loss of function (LoF), gain of function (GoF) and gain of toxicity (GoT) are three major categories of phase separation pathogenic mechanisms in developmental disorders. The dysregulation of phase separation can influence chromosome maintenance, neuronal development and normal signaling pathway, which induces craniofacial anomalies, autism, intellectual disability and other developmental abnormalities.

Mutations result in decreased or eliminated protein LLPS capacity, which in turn leads to biological processes that are defective [30]. Deficiencies in LLPS can result in various pathological characteristics, while this section primarily explores the effects of the loss of function on chromosomal architecture and neuronal development. Studies have shown that the disruption of biological condensates can alter the structure of chromosomes, further dysregulate gene expression and lead to diseases. For example, methyl CpG binding protein 2 (MeCP2) is a key component of constitutive heterochromatin, which is critical for chromosomal maintenance and transcriptional silence [31]. Many mutations in MeCP2 can impair its ability to undergo LLPS, which is commonly observed in patients with Rett syndrome, a progressive neurodevelopmental disorder associated with severe mental disability and autism-like symptoms that predominantly affect girls during early childhood [ 32, 23] . Among the pathological mutations, the R168X mutant protein exhibits a significant reduction in its ability to partition into heterochromatin condensates, leading to changes in chromatin architecture **(** Figure 1 **)**. R168X mutant mouse embryonic stem cells also present evidence of Rett syndrome-associated cellular phenotypes [23]. Moreover, studies have reported that a minimal MeCP2 fragment (just containing methyl-DNA binding domain and NCoR-interaction domain) that retains condensate formation capability can partially prevent or reverse Rett syndrome phenotypes when introduced into MeCP2-deficient mice [ 23, 33] . These results further support the close correlation between the LLPS ability of MeCP2 and Rett syndrome. Another example of a protein involved in LLPS and associated with a developmental disorder is mixed lineage leukemia 4 (MLL4). MLL4 serves as a scaffold protein for transcriptional condensate nucleation, allowing for the recruitment of cofactors and activators through liquid-liquid phase separation [34]. Haploinsufficiency of KMT2D, which encodes MLL4, is mostly responsible for Kabuki syndrome, a rare multi-systemic disorder characterized by craniofacial anomalies, intellectual disability, and various organ malformations [35]. The LLPS ability of MLL4 depends on its intrinsically disordered region (IDR), which is conserved in multiple species and is specifically deleted in patients with Kabuki syndrome ( Figure 1) [ 36, 37] . Truncated mutations of MLL4’s IDR have been found to impair its LLPS ability and transcriptional condensates, altering the balance between transcriptional and Polycomb group (PcG) condensates, which are strong mediators of nuclear architecture [38]. This imbalance can lead to changes in the expression levels of genes that regulate chromatin architecture (such as *TOP2A* and *TOP2B*) [25], and it has been reported that restoring the expression levels of related genes can rescue the similar pathological features of Kabuki syndrome [ 25, 39] . These results suggest that the haploinsufficiency of MLL4 influences the expression levels of genes related to chromatin architecture, which could be the underlying cause of Kabuki syndrome.

Furthermore, condensate disruption during neuronal development can potentially have an impact on synaptic distribution and neuronal identity. For example, the transcription factor Tbx3 plays a critical role in establishing and maintaining the identity of KNDy neurons, which trigger puberty [29]. Mutations in TBX3 are linked to delayed puberty onset and ulnar-mammary syndrome (UMS), an autosomal dominant disorder that causes developmental issues [ 40, 41] . TBX3 undergoes phase separation to maintain the expressions of TAC3 and KISS1 in humans, which are important for shaping the identity and regulating the activity of KNDy neurons [29]. Pathological mutations in TBX3 disrupt its ability to undergo LLPS and significantly attenuate the transcriptional activation of TAC3 and KISS1 ( Figure 1), further interfering with the onset of puberty in UMS patients [29]. Autism spectrum disorders (ASD) are a group of neurodevelopmental disorders characterized by enduring and impairing social communication [42]. Cortactin-binding protein 2 (CTTNBP2) is a strong candidate for ASD that controls dendritic spine formation and maintenance [26]. Several mutations in CTTNBP2 that are linked to ASD have been shown to affect its LLPS ability. Specifically, the R533* mutation reduce the number of condensates compared to wild-type CTTNBP2 and also impact the synaptic distribution of the R533* mutant protein ( Figure 1) [27]. Mice carrying these mutations exhibit impaired social interaction behaviors similar to those observed in individuals with ASD [ 26, 28] . Interestingly, the synaptic deficits and the social impairment of the mutant mice can be partially improved by stabilizing the condensates of CTTNBP2 and other synaptic proteins [27].

### Gain of function

Gain of function denotes the possibility of activating or improving a protein’s capacity for phase separation as a result of mutations. Developmental diseases can be caused by the gain of LLPS, which result in disease-related pathways or reactions being hyperactivated or lead to the sequestration of RNAs, proteins, or both. The non-receptor protein tyrosine phosphatase (PTP) SHP2, encoded by *PTPN11*, plays a crucial role in normal development by mediating RAS-mitogen-activated protein kinase (MAPK) signaling [43]. Germline heterozygous mutations of PTPN11 are associated with Noonan syndrome (NS) in 50% of cases [44] and Leopard syndrome (NS-ML) in 90% of cases [45]. The activating mutations of PTPN11 are viewed as gain-of-function (GOF) mutations. While the wild-type SHP2 disperses throughout the cell, all SHP2 variants with disease-associated mutations form discrete puncta ( Figure 1) [24]. It was reported that the LLPS-promoting mutations of SHP2 lead to the hyperactivation of RAS-MAPK by increasing the phosphorylation levels of both MEK1/2 and ERK1/2, which may explain the pathogenesis of the NS and NS-ML [ 46, 47] . Another example is the pathogenic R636S mutation of the human RNA-binding motif protein-20 (RBM20), which is associated with dilated cardiomyopathy (DCM) and heart failure [48]. Unlike the wild-type SHP2 that diffuses throughout the cell, RBM20 originally and primarily displays prominent splicing speckles in the nucleus ( Figure 1) [ 49, 21] . In patients with DCM, the RBM20 _R636S_ granules significantly accumulate in the sarcoplasm and distribute on the myofibril Z-discs [21]. The RBM20 _R636S_ granule may result in the sequestration of actin alpha cardiac muscle 1, further disrupting the actin cytoskeleton of cardiomyocytes [ 21, 50] .

### Gain of toxicity

Different from the gain of function, protein gain of toxicity represents the mutation of the gene results in the acquisition of the aggregation propensity that can interrupt the normal function of the wild-type protein, which may lead to abnormal aggregation and cell toxicity. Similar to that in neurodegenerative diseases, the aggregation state of associated proteins is correlated with the dysregulation of physiological function [51]. For example, DDX3X is a prominent component of cytosolic RNA granules and participates in all facets of RNA metabolism [52]. DDX3X-related developmental delay/intellectual disability (ID) typically occurs in females and very rarely in males. The L556S missense mutation in DDX3X’s core helicase domain induces its conformational changes, exposing hydrophobic residues to the solvent and resulting in a high propensity for self-aggregation and production of amyloid-like assemblies ( Figure 1) [22]. These aggregates can sequester wild-type DDX3X protein and lead to cell toxicity, because even a 25% reduction in DDX3X levels can strongly deregulate neurogenesis [53]. In addition, more cases of neurodegenerative diseases that are caused by gain-of-toxicity will be shown in the next session.

## Phase Separation with Tandem Repeat Disorders

In addition to developmental disorders, phase separation also plays a critical role in tandem repeat disorders (TRDs). TRDs are caused by the abnormal expansions of short tandem repeats (STRs) that are 2‒12 base pairs long DNA repeating tracts and locate in both coding and non­coding regions. More than 50 human disorders are now known to be caused by STR expansion [ 54, 55] . These disease­causing repeat expansions can range from a few to thousands of repeats and reside within gene 5′ untranslated regions (UTRs), coding exons, 3′ UTRs or introns [56]. STR expansions induce a series of changes in molecular and cellular processes through either loss of function or gain of toxicity mechanisms at the DNA, RNA or protein levels ( Table 2). Loss-of-function mechanism includes expansion within non-coding region that induces transcription silencing, and expansion within coding region generates nonfunctional protein. However, there is little evidence that the mechanism involves phase separation.

**Table:** **Table 2** Phase separation and nucleotide expansion disease

Gene	Pathological type	Disease	Repeat sequence	Mechanism	Reference
*FMR1*	RNA gain of function	Fragile X-associated tremor/ataxia syndrome (FXTAS)	CGG	RNA-mediated recruitment of proteins attracted by CGG repeats in *FMR1* RNA foci.	[ 57, 58]
*DMPK*	RBP sequestration and RAN translation	Myotonic dystrophy type 1 (DM1)	CTG	The expanded *DMPK* RNA and MBNL1 are regulators of the formation and turnover of cytoplasmic SGs in DM1.	[59]
*CNBP*	RBP sequestration and RAN translation	Myotonic dystrophy type 2 (DM2)	CCTG	The expanded *CNBP* RNA and MBNL1 are regulators of the formation and turnover of cytoplasmic SGs in DM1.	[60]
*C9orf72*	RBP sequestration and RAN translation	Amyotrophic lateral sclerosis and frontotemporal dementia (ALS and FTD)	GGGCCC	Through multivalent base pairing alone, (GGGGCC)5 RNAs can form RNA droplets through phase separation *in vitro*.	[61]
*HTT*	polyglutamine gain of function	Huntington disease (HD)	CAG	The huntingtin exon1 proteins can form reversible liquid-like assemblies, which are converted to solid-like assemblies when poly Q abnormal expansion.	[62]
*ATXN1*	polyglutamine gain of function	spinocerebellar ataxias 1 (SCA1)	CAG	The aggregation of ATXN1 with expanded poly Q in neuronal processes can disrupt crucial cargo trafficking and trap other proteins.	[63]
*ATXN2*	polyglutamine gain of function	spinocerebellar ataxias 2 (SCA2)	CAG	ATXN2 has a C-terminal low-complexity domain (LCD), which regulates ATXN2 liquid-liquid phase separation. Expanded poly Q in ATXN2 alters stress granule dynamics and induces protein aggregation.	[ 64‒ 66]
*ATXN3*	polyglutamine gain of function	spinocerebellar ataxias 3 (SCA3), also known as Machado-Joseph Disease (MJD)	CAG	Poly Q expansion promoted ATXN3 self-assembly into insoluble SDS-resistant aggregates.	[ 67, 68]
*CACNA1A*	polyglutamine gain of function	spinocerebellar ataxias 6 (SCA6)	CAG	A C-terminal fragment of CACNA1A containing the polyQ tract remains soluble in normal brains, but becomes insoluble mainly in the cytoplasm of human SCA6 Purkinje cells.	[69]
*ATXN7*	polyglutamine gain of function	spinocerebellar ataxias 7 (SCA7)	CAG	Polyglutamine expansion in ATXN7 causes its misfolding and intranuclear accumulation, leading to changes in interaction proteins, resulting insoluble nuclear inclusions.	[70]
*TBP*	polyglutamine gain of function	spinocerebellar ataxias 17 (SCA17)	CAG	PolyQ expansions within TBP alter its cellular distribution and transcriptional activity. TBP becomes progressively insoluble as polyQ repeat length increases.	[71]
*AR*	polyglutamine gain of function	Spinal and bulbar muscular atrophy (SBMA)	CAG	The expanded polyQ tract severely affects AR transcriptional activity and increases AR aggregation.	[72]
*PABPN1*	Polyalanine gain of function	Oculopharyngeal muscular dystrophy (OPMD)	GCG	The RNA binding protein PABPN1 promotes the formation of NPAD through its N-terminal disordered domain and RNA-recognized motif by liquid phase separation.	[73]
*HOXD13*	Polyalanine gain of function	Type II synpolydactyly (SPD II)	GCG	I Synpolydactyly-associated repeat expansions enhance HOXD13 IDR phase separation and alter the transcriptional co-activators in HOXD13-containing condensates.	[74]
*HOXA13*	Polyalanine gain of function	Hand-foot-genital syndrome (HFGS)	GCG	The HOXA13 IDR facilitated phase separation and HOXA13 IDR droplets exhibited a liquid-like FRAP rate. The HOXA13 IDR containing a short (+7A) HFGS-linked expansion tended to form aggregates with negligible FRAP rate.	[74]
*RUNX2*	Polyalanine gain of function	Cleidocranial dysplasia (CCD)	GCG	The RUNX2 IDR containing a CCD-associated alanine expansion a (+10A) tended to form solid aggregates, while RUNX2 IDR droplets exhibited a liquid-like FRAP rate.	[74]
*NOTCH2NLC*	Polyglycine gain of function	Neuronal intranuclear inclusion disease (NIID)	GGC	GGC repeats embed into the open reading frame of a small protein (uN2C) and is translated into a uN2C polyglycine-containing protein (uN2CpolyG) in NIID.	[75]

A growing number of studies have discovered that aberrant phase separation is associated with gain-of-toxicity in STRs. For example, at RNA level, the premutation range of CGG repeat expansions in the 5′ UTR of *FMR1* gene can generate toxic RNA foci that are regulated by multivalent interactions, leading to intranuclear inclusion formation in fragile X-associated tremor and ataxia syndrome (FXTAS) [76]. At protein level, CAG repeat expansions in the exon of huntingtin ( *HTT*) gene promote a solid-like state and cause intracellular aggregation of HTT protein in Huntington’s disease (HD) [62]. In this section, we will briefly review the expanded repeat toxicity resulting in aberrant phase separation in TRDs.

### Formation of aberrant RNA foci

**Figure 2:**
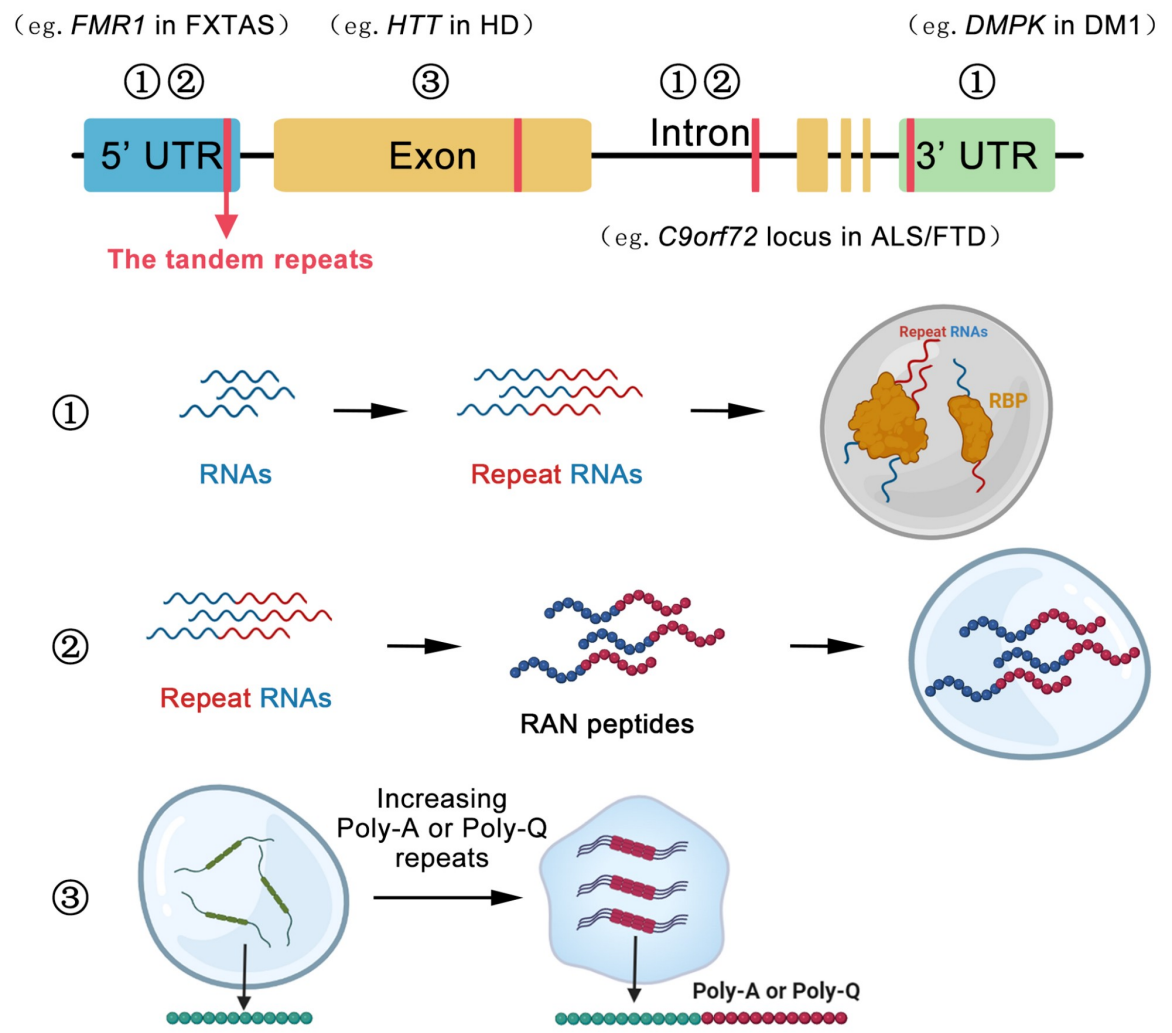
Figure 2 Molecular mechanisms of tandem repeat disorder pathogenesis (1) The tandem repeats can locate in UTR and intron, the tandem repeat RNAs may recruit RNA-binding proteins and cause the formation of RNA foci, such as the CGG repeats in the 5' UTR of FMR1, CTG repeats in the 3' UTR of DMPK and GGGGCC repeats in the intron of C9orf72 locus, which cause FXTAS, DM1 and ALS/FTD, respectively. (2) The repeat-associated non-AUG (RAN) translation of toxic peptides contributes to the pathogenesis of various TRDs, such as FMRpolyG encoded by FMR1 mRNA containing expanded CGG repeats and the PRn-poly-dipeptide encoded by C9orf27 repeat expansions. (3) Also, the CAG and GCG repeat expansions in the exon result in the formation of poly-Q and poly-A tracts in related proteins, respectively, that promote the formation of solid aggregates and cause neuronal dysfunction. For example, the CAG repeats in the exon of HTT cause the expression of polyglutamine (poly-Q) and cause Huntington disease (HD)

The formation of RNA foci is a classic example of an RNA-mediated gain of toxicity mechanism. This process is intricate and involves the formation of unusual secondary structures by repeat RNA products. Through Watson-Crick and non­canonical base pairing, repeat RNAs can form imperfect hairpin structures and G-quadruplexes. The GGGGCC expansion in the intron of *C9orf72* locus is the most frequently known cause of amyotrophic lateral sclerosis and frontotemporal dementia (ALS/FTD) ( Figure 2). GGGGCC expansions have been found to form G-quadruplexes both *in vitro* and *in vivo* [ 77‒ 79] . Repeat hairpins and G-quadruplexes are thought to facilitate RNA-RNA interactions. Through multivalent base pairing alone, 47×(CUG) and 5×(GGGGCC) RNAs can form RNA droplets through phase separation *in vitro*. The longer length of these expanded RNA repeats promotes the formation of gel-like or solid-like droplets [61]. As another example, Myotonic dystrophy type 1 (DM1) is a complex neuromuscular disorder caused by CTG repeat expansions in the 3′ UTR of DM1 Protein Kinas ( *DMPK*) gene [ 80‒ 82] ( Figure 2). Healthy individuals carry repeat tracts below 37 repeats, whereas DM1 patients usually carry more than 50 repeats. Expanded CUG RNAs likely form highly stable hairpin structures [ 83, 84] .

Besides repeat RNAs, RNA-binding proteins (RBPs) attracted by expanded repeat RNAs can also impair the dynamics of condensates and stabilize the formation of RNA foci [56]. In DM1, the expanded *DMPK* RNA foci in the cell nucleus recruit a multi-functional RBP—muscleblind-like splicing regulator 1 (MBNL1). The expanded *DMPK* RNA and MBNL1 are regulators of the formation and turnover of cytoplasmic SGs in DM1 [59]. Similarly, myotonic dystrophy type 2 (DM2) is caused by unstable CCTG repeat expansions in the intron of the CHC-type zinc finger nucleic acid binding protein ( *CNBP*) gene. The expanded *CNBP* RNAs and multi-functional RBP MBNL1 promote the assembly of ribonucleoprotein (RNP) granules or RNA foci in DM2 [60]. The formation of RNA foci is also the typical hallmark of FXTAS which is a progressive neurodegenerative disorder [76]. FXTAS is caused by the premutation range (55‒200×CGG) repeats of *FMR1* [ 85‒ 88] ( Figure 2). One of the main mechanisms to explain the onset and development of FXTAS is the RNA-mediated recruitment of RBPs complex attracted by CGG repeats in *FMR1* RNA. The RBPs complex includes DiGeorge syndrome critical region gene 8 (DGCR8), heterogeneous nuclear ribonucleoprotein (hnRNP) A2/B1 and src-associated substrate during mitosis of 68 kDa (Sam68). The RNA foci behave as ribonucleoprotein (RNP) condensates that phase separate in the nucleus, forming large ubiquitylated inclusions [89].

The toxic RNA foci in repeat expansion diseases are involved in the transition from soluble RNA to RNA-protein phase separation. This process is defined by the sum of RNA-RNA, RNA-protein and protein-protein interactions. New discoveries are expected to follow, which will further broaden our understanding of how phase separation is regulated and the detailed mechanisms of RNA toxicity in TRDs.

### RAN-translation generates toxic peptides

Moreover, it was found that repeat-associated non-AUG (RAN) translation of toxic peptides contributes to the pathogenesis of various TRDs. RAN translation represents the translation of tandem repeats into peptides consisting of repeating amino acid sequences that do not require AUG initiation [55]. Apart from RNA foci formation, repeat RNAs can recruit translation machinery and produce toxic peptides by RAN-translation [90]. Expanded *C9orf72* RNA aberrantly recruits translation machinery and expresses putatively toxic RAN translation products [ 91‒ 93] . Recent studies have shown that PR _n_-poly-dipeptide encoded by C9orf72 repeat expansions can bind to different low-complexity domain (LCD)-containing proteins, which in turn results in impaired functions of multiple membrane-less organelles [94] ( Figure 2). In FXTAS, another main mechanism is the aggregation of repeat-associated RAN polyglycine peptides [ 57, 58] . *FMR1* mRNA containing expanded CGG repeats initiates RAN translation and produces a polyglycine-containing protein, FMRpolyG ( Figure 2). The polyglycine region of FMRpolyG has low-complexity disordered domains with RNA binding ability. FMRpolyG can directly interact with CGG repeat-derived RNAs and undergo the liquid-to-solid transition, leading to FMR polyG aggregates [95]. In DM2, the role of RNA toxicity is well established, in which the RAN-translated peptides polyLPAC and polyQAGR are expressed in various brain regions [96]. Because RAN translation can produce peptides in a variety of reading frames [97], toxic peptides may contribute to dysfunction in various tandem repeat disorders.

### Expansion within coding regions generates toxic proteins

When the tandem repeats locate within an exon coding region, the resulting TRDs typically exhibit gain-of-function phenotypes, characterized by abnormal protein aggregation and phase separation [ 98, 99] . In repeat expansion diseases, aggregated proteins play a direct role in pathogenesis.

#### Polyglutamine tracts-mediated gain of toxicity

At least nine disorders, including Huntington disease (HD), several spinocerebellar ataxias (SCAs), and spinal and bulbar muscular atrophy (SBMA), are caused by CAG repeat expansions in coding sequences that result in the expressions of polyglutamine-containing proteins [100]. As follows, we will introduce several TRDs caused by CAG repeat expansions.

HD is an extensively studied polyglutamine TRD that is characterized by the expansion of a translated CAG repeat located in the N-terminus of the huntingtin (HTT) protein. Wild-type individuals contain 6–34 CAG repeats in the *HTT* gene, while HD patients contain 36–121 repeats ( Figure 2). The expanded CAG repeats disrupt the normal splicing of the *HTT* gene, leading to the production of huntingtin exon 1 protein that is encoded by the first exon of *HTT* gene and contains an abnormally polyQ region. Huntingtin exon 1 protein can form reversible liquid-like assemblies, a process driven by huntingtin’s polyglutamine tract and a proline-rich region. However, the aberrantly expanded polyglutamine promotes the liquid-like to solid-like assemblies with a fibrillar structure in neurons, leading to neuron death, especially neuronal dysfunction in the striatum [62].

Another main type of TRD with tandem repeated polyglutamine tracts in an exon is Spinocerebellar ataxias (SCAs). The clinical hallmark of all SCAs is progressive atrophy of the cerebellum, brainstem, and spinal cord [101]. Most SCAs (including SCA1, 2, 3, 6, 7, and 17) are caused by the expansion of a translated CAG repeat. For instance, spinocerebellar ataxia type 2 (SCA2) is attributed to the abnormal CAG expansion in ATXN2, an RBP which could regulate stress granule assembly and translation [102]. ATXN2 has a C-terminal LCD, which contributes to liquid-liquid phase separation. ATXN2 normally contains 22‒23 CAG repeats on the N-terminus. Intermediate-length (27‒33) CAG repeat expansions in ATXN2 act as risk alleles for ALS [103] and larger expansions (34 or more repeats), trigger protein aggregation, and cause SCA2 [ 64‒ 66] . Various cellular functions and cellular homeostasis can be compromised by the aggregation of polyglutamine disorder proteins in neural processes, which can interfere with important cargo trafficking [63] and trap other proteins [104]. In addition to RNA-binding proteins, transcription factors can also harbor polyglutamine expansion. The CAG repeat expansions in the androgen receptor (AR), a transcription factor that controls the development of the prostate, result in spinal and bulbar muscular atrophy (SBMA), an X-linked, adult-onset neuromuscular illness [105]. The N-terminal domain (NTD) is critical for efficient condensate formation [106]. The AR transcriptional activity is significantly impacted by the enlarged polyQ tract. SBMA’s pathophysiology has been connected to the accumulation of motor neuron-toxic AR-polyQ in the nucleus [72].

#### Polyalanine tracts-mediated gain of function

The repeat expansions in an exon that encodes polyalanine tracts instead of the polyglutamine tracts outlined above are frequently linked to severe developmental abnormalities like synpolydactyly, X-linked mental retardation, and muscular dystrophy [107]. Many of these repeat expansions in human disorders occur in IDRs of transcription factors (TFs). Disease-associated repeat expansions in TFs such as HOXA13, RUNX2 and HOXD13 have been found to alter their phase separation properties [74]. These diseases are associated with the propensity of the protein to form solid aggregates and to alter its subcellular localization [ 108, 109] .

The polyalanine tracts in IDR of these three typical TFs (HOXA13, RUNX2 and HOXD13) show varied effects on their phase separation ability, including both impairment and enhancement. Firstly, HOXA13 is a homeobox TF. The polyalanine repeat expansion from 18 to 24–26 in the N-terminal IDR of HOXA13 results in hand-foot-genital syndrome (HFGS) [110], a rare, dominantly inherited condition characterized by distal limb malformations and genitourinary tract defects [111]. While the wild-type HOXA13 IDR can undergo phase separation and form liquid-like droplets, the HOXA13 IDR with a short (+7A) HFGS-linked expansion tends to aggregate [74]. Secondly, RUNX2 is a RUNT family TF that controls bone morphogenesis and expansions of a short alanine and glutamine repeat in the RUNX2 IDR. GCG repeats expansion in RUNX2 IDR is associated with cleidocranial dysplasia (CCD), a rare autosomal dominant disorder of severe skeletal defects [ 112, 113] . The RUNX2 IDR containing a CCD-associated alanine expansion a (+10A) tends to form solid aggregates, while RUNX2 IDR droplets exhibit liquid-like dynamics [74]. The pathological alanine repeat expansion alters its phase separation capacity, co-condensation with the MED1 IDR, and transcriptional activity. Another similar example is the alanine repeat expansions in the IDR of HOXD13, which cause type II synpolydactyly (SPD II) in humans [114].

In addition to transcription factors (TFs), alanine repeat expansions also occur in polyA binding proteins. PABPN1 is an abundant nuclear protein that binds with high affinity to nascent polyA tails. The RNA binding protein PABPN1 promotes the formation of nuclear polyA domains (NPADs) through its N-terminal disordered domain and RNA-recognized motif by liquid phase separation [73]. Expansion of GCG repeat from the normal 6 copies to 8‒13 copies leads to autosomal dominant oculopharyngeal muscular dystrophy (OPMD) disease [115]. In OPMD muscle models, alanine-expanded PABPN1 accumulates in insoluble intranuclear inclusions (INIs) and also abnormally accumulates in the cytoplasm [116].

#### Polyglycine tracts-mediated gain of function

In addition to polyglutamine and polyalanine disease, polyglycine (polyG) disease is defined as a novel class of TRDs recently. Expansion of GGC repeats in the 5′ UTR of the *NOTCH2NLC* ( *N2C*) gene causes neuronal intranuclear inclusion disease (NIID), which is a neurodegenerative disease characterized by the presence of intranuclear inclusions [75]. GGC repeats embed into the open reading frame of a small protein (uN2C) and is translated into a uN2C polyglycine-containing protein (uN2CpolyG) in NIID [ 75, 117] .

Altogether, short tandem repeats may alter the structure and function of proteins, differentially engage with their interacting partners and result in abnormal phase separation in the form of intracellular inclusions, and finally lead to neurological and muscular disorders cord [101].

## Aberrant Phase Separation and Cancer

In addition to the tandem repeat disorders and developmental disorders, aberrant MLOs have also been linked to a range of cancers by disrupting tumor suppression and normal signal transduction pathways, hyperactivating oncogenic genes or affecting protein quality control machinery. In this part, we will discuss tumorigenesis in relation to abnormal phase separation ( Table 3).

**Table:** **Table 3** Protein phase separation and cancer

Protein	Pathological type	Cancer	Mechanism	Reference
DnaJB1-PKAcat	Gain of function	Oncocytic pancreatic and biliary neoplasms, fibrolamellar hepatocellular carcinoma	DnaJB1-PKAcat suppresses the phase separation of RIα and leads to signal transduction disorder.	[ 118, 119]
EWS-FLI1	Gain of function	Ewing Sarcoma	EWS-FLI1 condensate recruits BRG1–BRM-associated factor (BAF) chromatin remodeling complex to upregulate the cancer-associated gene expression.	[120]
NUP98-HOXA9	Gain of function	Myelodysplastic syndromes and acute myeloid leukemia	NUP98-HOXA9 condensate promotes its chromatin occupancy and upregulates leukemogenic genes.	[ 121, 122]
EML4–ALK	Gain of function	Non-small cell lung cancer	EML4-ALK condensate enriches with RAS-activating factors (GRB2/SOS1/GAB1) and excludes the RAS activity negative regulators (GTPase-activating protein) to hyperactivate the oncogenic RTK/RAS signaling.	[123]
CCDC6-RET	Gain of function	Lung adenocarcinoma, thyroid gland papillary carcinoma, poorly differentiated thyroid gland carcinoma, breast invasive ductal carcinoma, and thyroid gland undifferentiated (anaplastic) carcinoma	CCDC6-RET condensate increases RAS signaling and MAPK signaling.	[ 123, 120]
PML- RARα	Gain of function	Acute promyelocytic leukaemia	PML-RARα disturbs the formation of PML bodies which function as tumor suppressors and triggers the formation of dispersed microspeckles and promote cancer development.	[ 124‒ 126]
SHP2	Gain of function	Juvenile myelomonocytic leukemias	Activating SHP2 mutants’ condensate triggers Ras-MAPK pathway hyperactivation. Inactivating SHP2 mutants’ condensate recruits the wildtype SHP2 to trigger Ras-MAPK pathway hyperactivation.	[24]
ENL	Gain of function	Wilms tumor and acute myelocytic leukemia	ENL mutants phase separates native target genes and drives oncogenic gene hyperactivation.	[127]
Androgen receptor (AR)	Gain of function	Prostate cancer	Antiandrogen treatment will promote the formation of transcriptional condensates formed by antiandrogen-resistant androgen receptor mutants.	[ 106, 128]
KDM6A/UTX	Loss of function	Acute myeloid leukemia, bladder carcinoma, breast cancer, chronic myeloid leukemia, colorectal adenocarcinoma, endometrial adenocarcinoma, and glioblastoma	UTX mutants lose phase separation capability which was associated with the cancer-suppressive properties.	[ 17, 129]
SPOP	Loss of function	Prostate cancer	SPOP mutants lose phase separation capability and have decreased ubiquitination activity, leading to substrate accumulation.	[130]
AKAP95	Loss of function	Triple negative breast cancer	AKAP95 mutants’ condensate was less dynamic and had decreased splicing and transcription regulation activity.	[131]
Axin and APC	Loss of function	Colorectal cancer	APC mutants lose phase separation capability and release β-catenin protein, leading to the hyperactivation of the Wnt pathway.	[ 132, 133]
Gα protein i2 (Gαi2)	Loss of function	Colorectal cancer	Gαi2 mutant can not induce the formation of axin2 condensate which promotes the degradation of β-catenin.	[134]
KAT8	None	Lung cancer	KAT8 undergoes phase separation and forms condensate with IRF1, enriching the transcription apparatus to promote tumor immune evasion.	[135]

### Abnormal condensates impair tumor-suppressive function

**Figure 3:**
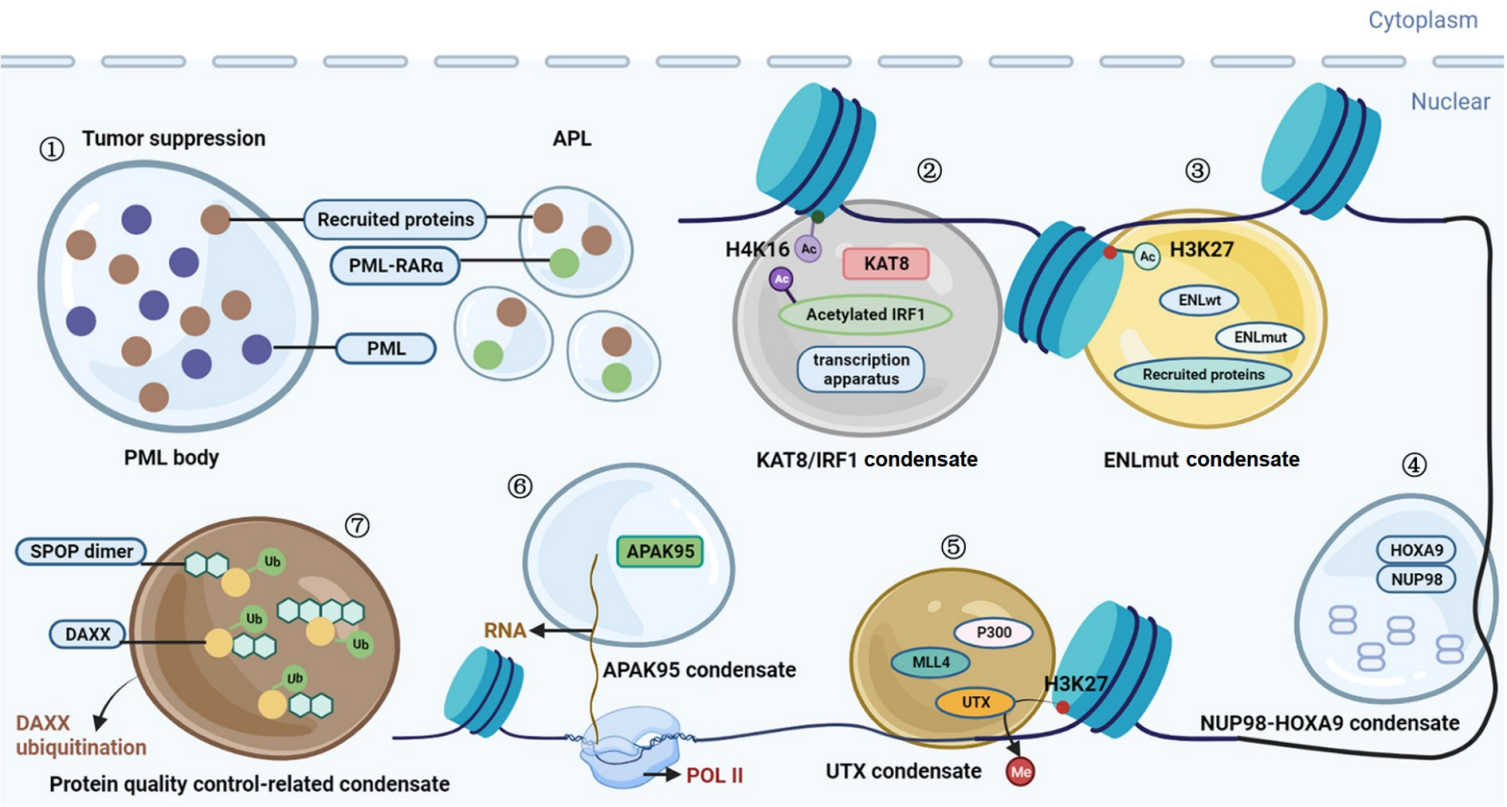
Figure 3 Phase separation and cancer Aberrant MLOs are linked to a range of cancers. (1) The disruption of promyelocytic leukemia (PML) nuclear bodies impairs the tumor suppressor function. (2) KAT8 acetylates IRF1 and forms condensate with PD-L1 and other transcription apparatus to increase PD-L1 expression, allowing cancer cells to evade immune surveillance. (3) ENL mutants form functional condensates with wild-type ENL to activate oncogenic gene. (4) NUP98–HOXA9 condensates promote its chromatin occupancy, contributing to the progression of leukemia. (5) Mutations in the UTX gene can disrupt the formation of tumor-suppressor condensates to dysregulated gene expression and promote cancer development. (6) AKAP95 forms phase-separated, liquid-like condensates in the nucleus and plays a crucial role in tumorigenesis. (7) Disruption the condensates of SPOP and DAXX could affect protein regulation, contributing to the development or progression of cancer.

Condensates are essential in preventing and restraining cancer development. They achieve this by assembling protein complexes involved in tumor suppression, and enhancing the expression of the related gene to suppress antitumor immune surveillance. However, the abnormal condensates will impair these tumor-suppressive functions. For instance, promyelocytic leukemia (PML) bodies are submicron-scale nuclear membrane-less organelles composed of proteins including the PML protein which function as tumor suppressors [ 136, 137] . PML protein contains a conserved RING finger/B-box/coiled-coil (RBCC) domain and a SUMO-interacting motif (SIM) [138]. The RBCC domain promotes the assembly of PML bodies, while the SIM interacts with sumoylated proteins, enhancing the assembly of PML bodies [138]. In acute promyelocytic leukemia (APL), a subtype of acute myeloid leukemia, the N-terminal of PML fuses with retinoic acid receptor alpha (RARα), resulting in the absence of the C-terminal SIM [124]. This fusion protein, PML-RARα, disturbs the PML bodies and triggers the formation of dispersed microspeckles, leading to the dysfunction of nuclear receptor-induced differentiation and PML-triggered apoptosis, which may aid the development of cancer [ 124‒ 126] ( Figure 3). Additionally, LLPS works to promote tumor immune evasion. In tumor cells, the histone acetyltransferase KAT8 undergoes phase separation and forms condensate with IRF1 upon induction by interferon-γ. KAT8/IRF1 condensation promotes IRF acetylation by binding to the promoter of PD-L1, which further enriches the transcription apparatus to upregulate PD-L1 [135] ( Figure 3). PD-L1 has been demonstrated to be a dominant suppressor of antitumor immune surveillance [ 139, 140] . Notably, based on the mechanism of KAT8/IRF1 condensate formation, a constructed competitive peptide disrupts condensate formation and consequently inhibits PD-L1 expression to enhance antitumor immune responses [135].

### Abnormal condensates impacting transcriptional functions

Abnormal condensates exert a significant influence on transcriptional processes, thereby driving the development and progression of cancer. These aberrant biomolecular assemblies can upregulate the expression of oncogenes, leading to cancer cell growth and survival. For example, ENL is a histone acetylation reader that uses its YEATS (Yes-associated protein) domain to recognize acetylated lysine residues [141]. Gain-of-function ENL YEATS mutations have been linked to acute myeloid leukemia (AML) and Wilms tumor, as they enhance transcriptional activation [ 142, 143] ( Figure 3). Recent research has shown that these mutations can trigger condensate formation at native target genes, even though the mutations are located in the structured YEATS domain. This, in turn, drives the hyperactivation of oncogenic genes [127]. EWS-FLI1 is a fusion protein that is generated from chromosomal rearrangement that is found in Ewing′s sarcoma, a rare type of cancer [144]. Through its low-complexity prion-like domain, EWS-FLI1 can undergo phase separation and interact with other proteins and DNA [ 145, 146] . Unlike the wild-type FLI1, the EWS-FLI1 droplet tightly binds GGAA microsatellites and recruits the BAF chromatin remodeling complex, leading to the upregulation of cancer-associated genes and the development of cancer [146]. NUP98-HOXA9 is another fusion protein commonly found in AML [147]. Due to the FG repeats from the N-terminus of NUP98, NUP98-HOXA9 can undergo phase separation and form condensates, which promotes chromatin occupancy and upregulates leukemogenic genes through a super-enhancer-like binding pattern [ 121, 122] ( Figure 3). In more detail, NUP98-HOXA9 condensate induces the formation of aberrant chromatin loops at protooncogenes-rich regions often without the assistance of CTCF [122]. In addition, transcription-related proteins can form condensates to upregulate cancer-suppressive genes, and certain site mutations may disrupt the formation of condensates, resulting in transcriptional repression and ultimately leading to cancer. KDM6A, also known as UTX, is an H3K27 demethylase [148]. Recent studies have demonstrated that UTX phase separation is linked to tumor-suppressive properties [17]. UTX phase-separated condensate recruits KMT2D/MLL4 and p300, thereby increasing their enzymatic activity at enhancers and upregulating tumor-suppressive genes [17] ( Figure 3). Specific mutations in the IDR of UTX may impair its phase separation capacity and cancer-suppressive function and replacing the IDR by another IDR can restore the phase separation capacity and tumor-suppressive function [17]. Furthermore, the dynamics of mRNA splicing and transcription-related condensates may also be important for cancer development. AKAP95 (A-kinase anchor protein 95) can regulate mRNA splicing and transcription. When overexpressed, AKAP95 can form condensates and is consistently linked to ovarian, rectal, and breast cancers [131]. However, tyrosine-to-phenylalanine mutations in the 101–210 region of AKAP95 result in the formation of more solid condensates with reduced biochemical reaction kinetics. Those mutations also impair the tumor-supporting abilities of AKAP95 while inhibiting the suppression of oncogene-induced senescence [131] ( Figure 3).

### Abnormal condensates disrupt signal transduction

In addition to dysregulated gene expression, abnormal MLOs can also affect signal transduction related to cancer. Recent papers have shown that mutant protein-formed condensates result in abnormal signal transduction and contribute to tumorigenesis. One such example is the non-receptor protein tyrosine phosphatase SHP2, which was mentioned above in Noonan syndrome and Leopard syndrome. Certain mutations result in hyperactivated RAS-MAPK pathway by activating SHP2 enzymatic activity or by recruiting and activating wild-type SHP2 protein, and ultimately cause malignancies [ 46, 47] . Additionally, fusion proteins may also form abnormal condensates that disrupt normal signal transduction pathways and contribute to cancer. One example is the EML4-ALK (echinoderm microtubule-associated protein-like 4–anaplastic lymphoma kinase) fusion protein, which is resulted from chromosomal rearrangements and is frequently detected in lung cancer [ 149, 150] . ALK is a receptor tyrosine kinase (RTK) that, in the chimeric protein, retains its intracellular kinase domain but loses its native transmembrane domain, while EML4 retains its trimerization domain and truncated TAPE domain [123]. EML4-ALK can undergo phase separation to form condensates in the cytoplasm, which are enriched with RAS-activating factors (GRB2/SOS1/GAB1) and exclude RAS activity negative regulators (GTPase-activating protein). This process is critical for the hyperactivation of oncogenic RTK/RAS signaling, which can contribute to the development of cancer [123]. Another example is the CCDC6-RET (coiled-coil domain containing 6- rearranged during transfection), another chimeric RTK oncoprotein which also promotes cancer development [ 151, 152] . Like EML4-ALK, CCDC6-RET can undergo phase separation driven by a coiled-coil domain and form cytoplasmic condensates that increase RAS signaling and downstream MAPK signaling [123]. Furthermore, apart from the hyperactivation of oncogenic signaling, disruption of normal condensates-mediated signal transduction by other proteins can also lead to cancer. RIα, a regulatory subunit of protein kinase A (PKA), forms functional condensates that determine signaling specificity [118]. In fibrolamellar carcinoma, the phase separation of RIα is suppressed by the fusion protein DnaJB1-PKAcat, leading to signal transduction disorder and uncontrolled hepatocyte growth [118]. Aside from proteins and nucleic acids, glycogen is another biomacromolecule that can undergo phase separation, as recently discovered [153]. Glycogen has been found to accumulate in liver tumor cells [154]. The condensate formed by this glycogen accumulation is enriched with the Laforin-Mst1/2 complex, which strongly inhibits the kinase activity of Mst1/2 and disrupts the formation of the WW45-Mst1/2 complex. This ultimately leads to the activation of oncogenic YAP signaling, which promotes cancer cell survival and transformation [153].

### Abnormal condensates interfere with protein degradation

Apart from influencing transcription and signal transduction, mutations in proteins may impact the formation of condensates that are associated with protein degradation, potentially contributing to the development of cancer. SPOP (speckle-type POZ protein) is a protein that belongs to a family of enzymes called E3 ubiquitin ligases [155]. Its mutant is always associated with solid tumors [156]. SPOP could undergo phase separation and form condensate with its substrate DAXX (death-domain-associated protein), which functions as active ubiquitination compartments to ubiquitinate DAXX [130]. However, certain cancer-associated mutants have lost their ability to bind with substrates, and consequently fail to undergo phase separation, leading to a significant reduction in ubiquitination activity, which results in substrate accumulation and contributes to cancer development [130] ( Figure 3).

## Phase Separation and Infectious Diseases

Abnormal protein phase separation is closely associated with several non-communicable diseases. Recent studies, however, have shown that protein phase separation also plays a significant role in the emergence of some infectious diseases, particularly viral disorders. For most viruses, phase separation is involved in the majority of viral lifecycles, including protein synthesis, genome assembly, virus assembly, budding and release. For example, the 52-KDa protein of human adenovirus regulates viral assembly by phase separation, and failure to form condensates results in failed packaging and assembly of only non-infectious particles. In contrast to non-communicable diseases, LLPS in viral diseases occurs in two distinct ways. It can either be employed by the host as a defense mechanism against invading pathogens, or it can be utilized by the pathogen to aid in their invasion [ 157‒ 159] .

### Phase separation promotes innate immunity to combat infection

Innate immunity is a non-specific immune response and is also the first line of defense against pathogens. Upon recognizing the pathogen, host cells initiate a cascade of events to activate the cells to attack and kill the pathogens. The cyclic GMP-AMP synthase (cGAS, cGAMP synthase) is an enzyme that initiates the innate immune response after detecting double-stranded DNA (dsDNA), either from the pathogen or self, in the cytoplasm [ 160, 161] . It converts GTP and ATP to cGAMP, a second messenger that activates the protein STING [ 160, 162] . This induces the synthesis of type I interferon and activates the NF-κB pathway [163]. cGAS directly recognizes dsDNA to undergo phase separation and forms a condensate in the cell, which is important for cGAS activation [ 9, 164] . However, pathogens have also evolved strategies to limit the formation of cGAS condensates. For example, ORF52 from gamma-herpesvirinae and VP22 from alpha-herpesvirinae, both belonging to the viral tegument protein family, can disrupt DNA-induced cGAS condensate formation in the early stages of infection [ 165‒ 167] . Another research group identified a spherical ER membranous condensate formed by ER-resident STING in the cell infected by a DNA virus or treated with cGAMP. This condensate recruits TBK1 (TANK binding protein 1), similar to the “STING-TBK1-cGAMP sponge”, to prevent innate immunity from overactivation [168].

### Viruses infect by impairing or utilizing stress granules (SGs)

**Figure 4:**
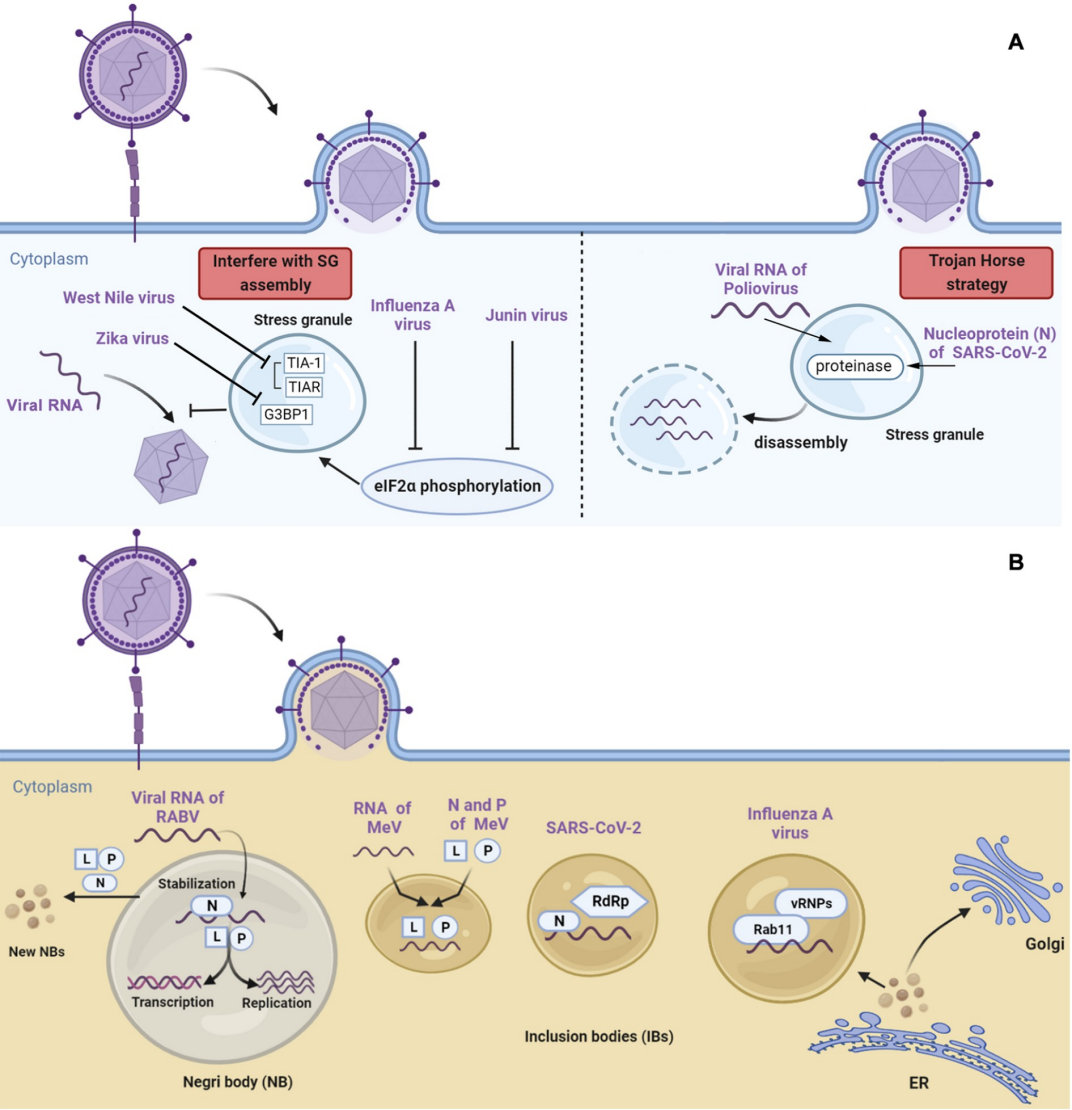
Figure 4 Phase separation in infectious diseases (A) Stress granule (SG) and Viral infection. The virus can suppress the formation of SGs to promote viral infection (left), also can evade host immunity using the Trojan Horse strategy (right). (B) Phase separation was exploited by the virus. The virus form liquid inclusion bodies (IBs) via LLPS and contribute to viral replication and even viral packaging.

SGs are considered as a defense mechanism against various stresses, which can sequester certain transcripts and proteins from the soluble portion of cytoplasm during physiological stress [ 12, 169] . In the context of viral infections, the formation of SG is also considered an antiviral strategy [170], but viruses have also developed ways to interfere with SG assembly [ 10, 170] ( Table 4). For example, the West Nile Virus can successfully infect cells by suppressing SG formation [ 178, 179] . The virus does this by blocking the TIA-1 and TIAR proteins, which are the scaffold proteins of SG, through its negative strand 3′ terminal stem-loop structure, disrupting SG [171] ( Figure 4A). Host cells that lack TIAR show compromised virus replication. Zika virus also limits SG formation by interacting with SG core proteins, and it even hijacks G3BP1 to facilitate viral RNA synthesis [177] ( Figure 4A). Junin virus uses an alternative mechanism to prevent SG assembly by inhibiting eIF2α phosphorylation, which is necessary for SG formation [173]. Moreover, Junin virus nucleoprotein and glycoprotein precursor can also interact with SG components to interfere with SG formation [173]. Influenza A virus follows a similar strategy, with its nonstructural proteins directly interacting with protein kinase R to block its kinase activity, which prevents eIF2α phosphorylation and suppresses SG formation [177]. In addition to interfering with SG formation, viruses can also use the Trojan Horse strategy to evade host immunity. For example, during the initial infection of Poliovirus, SGs enriched with viral RNA are formed, excluding G3BP-1, PABP, and eIF4G. These proteins are then cleaved by a proteinase expressed by the viral genome, named 3C proteinase, leading to the disassembly of SG [172]. Similarly, the SARS-CoV-2 nucleoprotein (N) can be recruited into SGs to block the interaction between G3BP1 and other core SG components, leading to SG disassembly [ 174‒ 176] ( Figure 4A). N protein-formed condensates can also inhibit the formation of MAVS (mitochondrial antiviral-signaling protein) condensates, downregulating the cytosolic IKK and TBK1 kinase activity, failing to activate the transcription factors IRF3 and NF-κB. This chain of events hampers the necessary upregulation of IFN1, thus impairing the initiation of innate immunity, which is key for fighting infections [180].

**Table:** **Table 4** Viruses fight with host phase separation-mediated antiinfection

Virus	Targeted MLOs	Mechanism	Reference
West Nile virus	Stress granule	Viral RNA binds with the host’s TIA-1 and TIAR to disrupt the assembly of stress granules.	[171]
Poliovius	Stress granule	Viral RNA enters into stress granules to exclude G3BP-1, PABP, and eIF4G.	[172]
Junin virus	Stress granule	Its protein inhibits eIF2α phosphorylation to disrupt the assembly of stress granules.	[173]
SARS-CoV-2	Stress granule	SARS-CoV-2 nucleoprotein interacts with G3BP1 and causes the disassembly of stress granules.	[ 174‒ 176]
Zika virus	Stress granule	Its protein interacts with SG core proteins to limit the formation of stress granules.	[177]
Influenza A virus	Stress granule	Its nonstructural proteins block kinase R activity, causing the dephosphorylation of eIF2α, leading to the disassembly of stress granules.	[177]

### Phase separation exploited by the virus for replication and assembly

Phase separation is a process that has been exploited by viruses. In addition to countering host condensate-based antiviral cell signaling, viruses have developed various strategies to take advantage of condensates enriched with both host and viral proteins for their replication, assembly and trafficking ( Table 5). During viral infection, viral liquid inclusion bodies (IBs) form inside cells, which are composed of viral proteins, nucleic acids, and other biomolecules that are critical for viral replication. For example, rabies virus (RABV) belongs to the order of Mononegavirales and possesses a single-stranded negative RNA genome [197]. Recent papers have shown that RABV is present in the cytoplasm of the infected neuron as liquid IBs, also known as Negri bodies (NBs), enriched with all the ribonucleoparticle (RNP) components, including nucleoprotein (N) protein, large (L) protein, and the phosphoprotein (P) [ 198‒ 201] . The N protein binds to viral RNA to stabilize it, while L and P proteins are responsible for the viral RNA transcription and replication [ 181‒ 183] ( Figure 4B). Researchers have also found that the RNPs of RABV could be excluded from NBs and transported via the microtubule network, with free RNPs able to assemble new virions and form new NBs [ 183, 198, 201] . Measles virus (MeV) also uses a similar replication mechanism, with N and P proteins undergoing phase separation and forming liquid-like condensates (or IBs) enriched with viral RNA [ 184, 185] . The formation rate of nucleocapsid correlates with the phase separation of N and P proteins, suggesting that viral replication is closely linked to viral protein phase separation [184] ( Figure 4B). Human respiratory syncytial virus (RSV) utilizes intracellular bodies (IBs) for RNA replication, which compartmentalize the N, P, L, and M2-1 proteins responsible for RNA replication [ 186, 187] . Researchers have identified two small molecules, the steroidal alkaloid cyclopamine and its analog A3E, which can disrupt the dynamics of IBs and suppress RSV replication [188]. Besides conventional IBs, another type of condensate called IB-associated granules (IBAGs) have been found in RSV-infected cells, which are enriched with phosphorylated M2-1, viral mRNA, and host phosphatase PP1 [ 189, 190] . In the cytosol, M2-1 is phosphorylated to unload its cargo, the mature polyadenylated mRNA [190]. The phosphorylated M2-1 is then recruited into IBAGs and dephosphorylated by PP1. The dephosphorylated form has a high affinity with newly synthesized viral mRNAs, protecting them and facilitating their polyadenylation [190]. In addition, phase separation is not only used by viruses to form IBs for viral RNA replication and maturation, but also for assembly and trafficking purposes. For example, Influenza A virus (IAV) forms liquid IBs with the host protein Rab11, concentrating the viral ribonucleoprotein (vRNP) complex and RNA close to the endoplasmic reticulum (ER) exit sites, promoting virion assembly and trafficking between the ER and Golgi [191] ( Figure 4B). Similarly, human immunodeficiency virus 1 (HIV-1) utilizes phase separation of its nucleocapsid protein (NC) to package the viral genome into the capsid and facilitate RNA trafficking. Disrupted nucleocytoplasmic transportation was observed when phase separation was impeded by chelating zinc ions [ 192‒ 194] . In SARS-CoV-2, the phase separation of N protein not only helps the virus evade host innate immunity but also potentially facilitates viral RNA transcription and replication through the recruitment of viral RNA-dependent RNA polymerase (RdRp) and viral mimic RNA [ 195, 196] ( Figure 4B). Additionally, the phase separation of N protein may play a role in viral packaging [ 202, 203] , with the characteristic of phase separation determined by the binding RNA and structure [203]. Changing the ratio of N protein and viral RNA can determine whether the mixture undergoes phase separation or dissolves, potentially affecting the organization of the long genome RNA or facilitating packaging into virions [203].

**Table:** **Table 5** Viruses exploit phase separation

Virus	Targeted MLOs	Mechanism	Reference
Rabies virus	Negri bodies (NBs)	NBs enriched with and stabilize all the ribonucleoparticle components responsible for viral replication.	[ 181‒ 183]
Measles virus	Inclusion bodies (IBs) and N and P proteins phase separation	IBs enrich with viral RNA. Viral replication depends on viral N and P proteins phase separation.	[ 184, 185]
Human respiratory syncytial virus	Inclusion bodies (IBs) and IB associated granules (IBAGs)	IBs compartmentalize N, P, L and M2-1 for RNA replication. IBAGs recruit and dephosphorylate M2-1 protein to protect and polyadenylate viral RNA.	[ 186, 187, 190]
Influenza A	Inclusion bodies (IBs)	The IBs concentrate viral ribonucleoprotein and RNA and promote the assembly of virion.	[191]
Human immunodeficiency virus 1	nucleocapsid protein phase separation	Facilitating viral RNA trafficking.	[ 193, 194]
SARS-CoV-2	N protein phase separation	N protein condensate recruits viral RNA-dependent RNA polymerase and RNA to promote viral RNA transcription and replication. N protein condensate may also play a role in viral packaging.	[ 195, 196]

### Phase separation: dual role in virus infections

Occasionally, phase separation plays a dual role during viral infections, serving both advantageous and disadvantageous functions depending on the specific situations and interactions at play. On one hand, phase separation is beneficial for the virus infection process. The p26 movement protein from the Pea enation mosaic virus 2 (PEMV2) undergoes phase separation and forms droplets within the host cell [204]. This concentration of proteins and other viral components facilitates systemic virus movement within the plant. Interaction with a host protein, fibrillarin (Fib2), appears essential for this process, suggesting that the virus exploits cellular processes for its replication. On the other hand, phase separation can be detrimental to the virus. The host plant can upregulate the expression of the RNA-binding protein G3BP under stress, leading to the formation of stress granules. This upregulation of G3BP, and the subsequent phase separation, restricts PEMV2 RNA accumulation, demonstrating an antiviral response. This highlights that host cells can use phase separation as a defense mechanism to limit viral replication. In summary, phase separation can be both beneficial and detrimental to viral infection, depending on the specific viral and host factors involved, reflecting the complex nature of virus-host interactions.

## The Potential LLPS-Associated Therapeutic Strategies

In recent years, it has been discovered that concentrating only on individual molecules may not be able to fully explain the complex illness phenotypes, and that it is also challenging to make a beneficial contribution to disease treatment methods by changing the structure or function of a single protein or nucleic acid. The investigation of phase separation may provide complex disease mechanisms, and even open up exciting new avenues for therapeutic intervention. We have discussed in detail how abnormal phase separation can lead to developmental abnormalities, neurodegenerative diseases, cancers, and viral infection. However, in most occasions, the relationships between phase separation and pathogenesis are currently simply correlative rather than causative, and warrant further investigation. In the meantime, finding effective therapies for diseases caused by abnormal phase separation remain challenging. Here, we list several potential therapeutic strategies. (1) Develop small molecules that can change the conformation of mutant proteins to inhibit their abnormal phase separation. For example, the allosteric inhibitors of SHP2 attenuate the phase separation of mutant SHP2 by locking it in a closed conformation [24]. (2) Using small molecule drugs to dissolve abnormally phase-separated condensates. For example, cisplatin selectively changes super-enhancer DNA, where MED1 is concentrated and forms condensate, and exerts its anti-neoplastic effect by dissolving these condensates [ 205, 206] . (3) Specific degradation of abnormally phase-separated proteins using PROTAC, AUTAC, and ATTEC, to dissolve the aberrant condensates [ 207‒ 209] . Recently, a BRD4-targeting PROTAC molecule was shown to significantly reduce the BRD4 condensates [210]. (4) Using specific competitive peptides to disrupt condensates. A 2142-R8 blocking peptide could competitively bind with KAT8 to disrupt KAT8-IRF1 condensates, further enhancing antitumor immune responses [135].

## Conclusions

**Figure 5:**
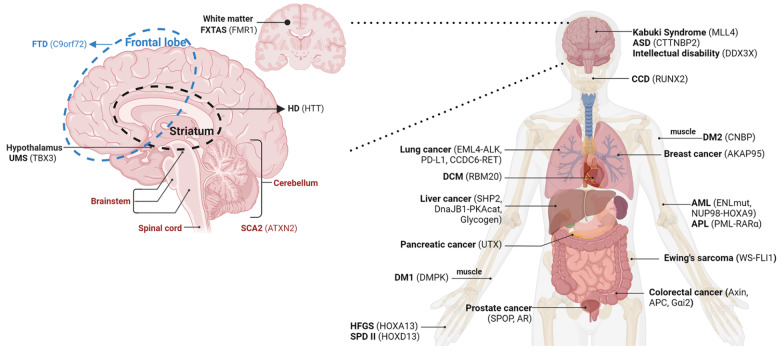
Figure 5 Onset location and causative protein of phase separation-associated non-infectious diseases Schematic diagram shows the location of the main disease-related proteins at the onset site.

In this review, we have laid out that phase separation is significantly correlated with various diseases and showed that both normal and abnormal phase separation can be related to disease onset. Aberrantly disrupted phase separation causes transcription dysregulation, chromatin architecture changes, low ubiquitination activity or impaired innate immunity. The mutants that gain LLPS ability can promote the condensate formation and lead to hyperactivating the disease-associated pathway or overexpressing the oncogenic genes. In addition, the fibrillar structure or the liquid-to-solid transition, which allows for the conversion into solid-like states, is the primary cause of many neurodegenerative illnesses. Further evidence that phase separation may be the primary cause of diseases is provided by the fact that LLPS-associated diseases are broadly spread across the entire human body, as illustrated in Figure 5. Therefore, phase separation is not only macromolecular membrane-less organelles with biological functions but also plays an important role in understanding and investigating the essence behind the disease, and even finding effective treatment strategies. As a result, phase-separated macromolecular organelles without a membrane play a crucial role in comprehending a variety of biological processes as well as the underlying causes of disease and even the development of effective therapeutic strategies.
